# An R package "VariABEL" for genome-wide searching of potentially interacting loci by testing genotypic variance heterogeneity

**DOI:** 10.1186/1471-2156-13-4

**Published:** 2012-01-24

**Authors:** Maksim V Struchalin, Najaf Amin, Paul HC Eilers, Cornelia M van Duijn, Yurii S Aulchenko

**Affiliations:** 1 Department of Epidemiology, Erasmus MC, Rotterdam, 3000 CA, The Netherlands; 2 Department of Biostatistics, Erasmus MC, Rotterdam, 3000 CA, The Netherlands; 3 Recombination and Segregation laboratory, Institute of Cytology and Genetics SD RAS, Novosibirsk, 630090, Russia

**Keywords:** single-nucleotide polymorphisms (SNPs), genome-wide association (GWA), gene-environment interactions (GxE), gene-gene interactions (GxG), variance heterogeneity, environmental sensitivity, VariABEL, the GenABEL project

## Abstract

**Background:**

Hundreds of new loci have been discovered by genome-wide association studies of human traits. These studies mostly focused on associations between single locus and a trait. Interactions between genes and between genes and environmental factors are of interest as they can improve our understanding of the genetic background underlying complex traits. Genome-wide testing of complex genetic models is a computationally demanding task. Moreover, testing of such models leads to multiple comparison problems that reduce the probability of new findings. Assuming that the genetic model underlying a complex trait can include hundreds of genes and environmental factors, testing of these models in genome-wide association studies represent substantial difficulties.

We and Pare with colleagues (2010) developed a method allowing to overcome such difficulties. The method is based on the fact that loci which are involved in interactions can show genotypic variance heterogeneity of a trait. Genome-wide testing of such heterogeneity can be a fast scanning approach which can point to the interacting genetic variants.

**Results:**

In this work we present a new method, SVLM, allowing for variance heterogeneity analysis of imputed genetic variation. Type I error and power of this test are investigated and contracted with these of the Levene's test. We also present an R package, VariABEL, implementing existing and newly developed tests.

**Conclusions:**

Variance heterogeneity analysis is a promising method for detection of potentially interacting loci. New method and software package developed in this work will facilitate such analysis in genome-wide context.

## Background

Genome-wide association studies (GWAS) have been instrumental in identifying genetic variants involved in complex diseases. In GWAS, the relation between a trait of interest and genetic variation (usually a single nuclear polymorphism -- a SNP) is studied by assessing hundreds of thousands of polymorphisms in thousands of individuals. Several hundreds of loci for dozens of complex human diseases and quantitative traits have been discovered using GWAS [1].

Though GWASs were successful in finding single loci associated with a trait, complex genetic models which include many interacting loci and environmental factors are of interest as they may help finding new loci and improve our understanding of the genetics of complex traits. A search for genetic interactions by direct analysis, in which all possible genetic models are examined, meets substantial computational and methodological difficulties. When millions of SNPs are considered, which nowadays has become routine in GWAS, testing for interaction for all possible pairwise combinations of SNPs becomes cumbersome requiring parallel computations using hundreds or thousands of CPU cores. Also, a large number of models has to be tested, resulting in multiple comparison problem, which weakens the statistical power and the possibility of new findings. For instance, if a simple interaction between two SNPs is considered in analysis of one million SNPs, approximately 5 · 10^11 ^unique SNP pairs are to be tested. This amount of tests is equivalent to running a standard "direct effects only" GWAS 5 · 10^5 ^times.

Thus, already the simple case of interactions between only two SNPs poses serious computational challenges. However, there is no reason why biology should not be more complex, involving more than two interacting variants. In general, a trait can be determined by a complex network of multiple interacting genes and other factors, including environmental ones. Statistical modeling of such complex network of interacting factors in genome-wide context would be a big challenge both methodologically and computationally. Prior information on loci, which are likely to be involved in a trait's control (e.g. genes in pathways implicated for specific trait) can help reducing the space of models to be tested but still does not solve the problem. For example, the protein pathway involved in Alzheimer's disease incorporates hundreds of genes. Each of them may include over 25-50 SNPs.

Another approach to dissection of genetic interactions consist of identification of potentially interacting loci, with further search for factors which interact with these loci. For quantitative outcomes interaction of a SNP with an unknown factor can be discovered from the trait's distribution conditional on the genotype: it is expected that trait will have larger variance for an interacting genotype [2,3]. This assumption can be tested using a variance heterogeneity test. Such testing is easily implemented and can be performed for the whole genome in a reasonable time. It also deals efficiently with the multiple comparisons problem, as the number of models to be tested in such analysis equals to the number of SNPs regardless of the complexity of the interaction model underlying the trait. In that, the variance test is similar to the regular GWAS (where the effect of a SNP on the phenotype mean is being studied). Methodologically, this approach has resemblance to the "environmental sensitivity" analysis [4,5].

Two groups [2,3] demonstrated that testing variance heterogeneity in GWAS is a promising approach for finding new genes involved in interactions. However the approaches proposed up until now cannot deal with imputed SNPs. Imputations are crucial for GWAS because they not only increase power in the analysis of an individual study, but also allow subsequent meta-analysis of the obtained results.

In this work we present a method extending variance heterogeneity analysis to imputed genetic data. We also develop VariABEL - an R package implementing variance heterogeneity tests proposed previously and developed in this work.

## Implementation

Here we describe existing variance heterogeneity tests and the newly proposed test, which is suitable for the analysis of imputed genetic data (subsection "Variance heterogeneity tests"). Next, we describe the setup of the simulations, which were used to study statistical properties of the new test (subsection "Simulations") and outline the details of implementation of our software (section "The VariABEL package").

### Variance heterogeneity tests

For measuring variance heterogeneity we have implemented two tests: Levene's test [6,7] and the test where linear regression is performed on squared residual values of a trait (Squared residual Value Linear Modeling, SVLM).

Levene's (the Brown-Forsythe) test is defined as:

(1) $$T^{2}=\frac{\left(N-k\right)\sum_{j=1}^{k}n_{j}{\left(Z_{j}.-Z..\right)}^{2}}{\left(k-1\right)\sum_{i=1}^{N}\left(Z_{i}-Z_{g_{i}}.\right)},$$

where *Z*_*i *_*= |y*_*i *_*- ỹ*_g*i*_| is the deviation of the value of the trait of *i*-th individual, *y*_*i*_, who has genotype *g*_*i *_from the median value of the trait in individuals having that genotype, *ỹ*_*gi*_*; N *is the total sample size, *n*_*j *_is the number of individuals with genotype *j, k *is the number of possible genotypes, $Z_{j}.=\frac{1}{n_{j}}\sum_{i=1}^{N}Z_{i}{I_{g_{i}}}_{=j}$ is mean deviation from the median for individuals having genotype *j *($I_{g_{i}=j}$ is an indicator variable which takes value of one if *g*_*i *_is equal to *j *and zero otherwise), and $Z..=\frac{1}{N}\sum_{i=1}^{N}Z_{i}$ is the mean deviation from the median across all individuals.

Under the null hypothesis of variance homogeneity, the value of the test statistic, *T*^2^, has an *F *distribution with *df*_1 _*= *(*k - *1) and *df*_2 _*= *(*N - k*) degrees of freedom. In a case of large *N, T*^2 ^is approximated well by the $\chi_{df=\left(k-1\right)}^{2}$ distribution. With three possible genotypes, *k - *1 = 2.

Genetic imputations routinely used in GWAS nowadays increase the power in the analysis of individual studies and also allow meta-analysis of the studies using different SNP arrays. In case of imputations the posterior probability of a genotype is estimated for each subject for a given SNP. Because standard variance heterogeneity tests assume that an observation should be known to belong to a certain group (i.e. an individual is known to have specific genotype with full confidence), they can not be directly applied to the imputed data.

To allow for variance heterogeneity test for imputed SNPs we propose a simple procedure (SVLM) described below. It is known from elementary statistics that by definition the variance is:

(2) $$\text{Var}\left(Y\right)=E\left[{\left(Y-E\left[Y\right]\right)}^{2}\right]=E\left[Y^{2}\right]-E{\left[Y\right]}^{2}$$

where *Y *is a random variable, Var(*Y*) is the variance of *Y*, *E*[*Y*] and *E*[*Y*^2^] are expected values of the variable *Y *and *Y *^2 ^correspondingly. In our case *Y *is a trait. The variance of *Y *conditional on the genotype *g *is *V *(*Y *|*g*) = *E*[(*Y *- *E*[*Y *|*g*])^2^|*g*]. This means that for each genotype the variance is equal to the mean of the squared residual of the trait conditional on the genotype.

**Figure 1:**
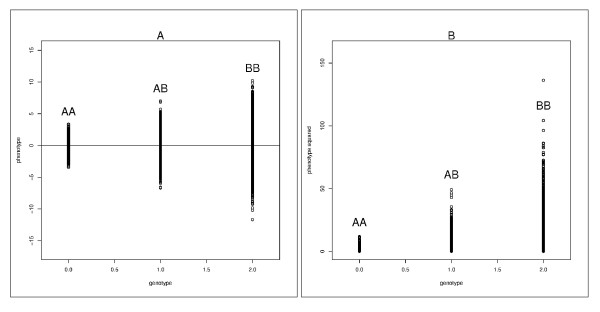
**Explanation of the SVLM test**. Biallelic SNP genotypes *(AA, AB, BB) *are presented on the X-axes. The *B *allele is in interaction with some factor. This interaction increases the variance of the trait when the number of *B *alleles present in the genotype increases. (A) Relation between the genotype and the value of the trait (B) Relation between the genotype and the squared residuals of the trait, conditional on the genotype.

To explain this idea we provide Figure 1. Panel 1A shows the relation between the trait value and the number of *B *alleles in the genotype. It is assumed that allele *B *is interacting with some quantitative factor, hence the variance of the trait is increasing as the number of *B *alleles, present in an individual's genotype, increases. Figure 1B shows the same data, but the points correspond to the squared residuals after subtracting genotypic mean from the trait's value. The means of these squared residuals in each genotypic group shown in panel B is equal to the variance within genotypic groups shown in panel A. Thus, taking squared residuals conditional on the genotype changes the task of estimation of the conditional variances into the task of estimation of the conditional means, which can be approached with using conventional methods such as regression analysis. Important covariates having large effects on means, can be easily accommodated in the model if necessary by modifying the expression used to compute the conditional mean.

Technically, the SVLM method consists of two steps. First, a regression analysis is applied where the trait is adjusted for a possible SNP effect and other covariates. Second, a regression analysis is applied to the squared values of residuals obtained from the first stage, using the SNP as the predictor.

### Simulations

To study Type I error and power of the SVLM test, we performed a simulations study. Similar to our previous work [3], we simulated the trait under following linear model

(3) $$y_{i}=\mu +\beta_{g}g_{i}+\beta_{F}F_{i}+\beta_{gF}\cdot g_{i}F_{i}+\varepsilon_{i},$$

where *y*_*i *_is the value of the trait for *i*^*th *^individual, *μ *is the intercept, *β*_*g *_is the direct effect of the SNP, *β*_*F *_is the direct effect of the interacting factor, *β*_*gF *_is the effect of interaction between the SNP and the factor, *g*_*i *_~ *B*(*n*_*g*_*,P*_*B*_) is a SNP, which is assumed to be binomially distributed with *n*_*g *_*= *2 (number of alleles in the genotype) and *P*_*B *_∈ [0; 1] (frequency of the interacting *B *allele). $F_{i}\sim N\left(\mu_{F},\sigma_{F}^{2}\right)$ is a factor, which is assumed to be normally distributed with mean *μ*_*F *_and variance $\sigma_{F}^{2}$. *ϵ*_*i *_is the random error, which follows a normal distribution with a zero mean and a variance of one. We assumed that the distributions of *g*_*i*_*, F*_*i*_, and *ϵ*_*i *_are independent. For our simulations, without loss of generality we can assume that *μ *= *μ*_*F *_*= *0, and $\sigma_{F}^{2}=1$.

Without loss of generality for both type I error and power the SNP effect was set to zero *β*_*g *_*= *0. We studied four different frequencies of the interacting allele: 5%, 40%, 60% and 95%. Results for 40% are presented in the text below. Results for other frequencies are shown in Additional file 1, Figure S1, Additional file 2, Figure S1 and Additional file 3, Figure S1. From the GWAS it is known that the regression analysis may lead to spurious results when the frequency of the minor allele is very low.

Therefore additionally we have studied type I error of the SVLM test for allele frequencies 0.0005, 0.00075, 0.001, 0.002, 0.003, 0.004 and 0.005. As SVLM requires squaring of the trait's residuals, the presence of extreme values can affect the type I error and power of this test. To check this, we studied three types of distribution of the residual error term *ϵ*_*i*_: one normal distribution and two types of *χ*^*2 *^distributions with degrees of freedom *df *= 1 and *df *= 5 respectively. We simulated data for 10000 individuals.

For studying Type I error effect of the factor and the effect of interaction term were both set to zero (*β*_*F *_= *β*_*gF *_= 0). Twenty thousands simulations were performed.

For studying dependence between power and the interaction effect, 1000 simulations were done under each simulation scenario. As we demonstrated previously [3], the magnitude of the genotypic variance difference and hence the power of the variance heterogeneity test depends not only on the effect of interaction between the genotype and the environmental factor, but also on the magnitude of the main effect of the interacting factor *F*. This dependence is not monotonic and, given other parameters are fixed, there is a certain optimal main effect of the factor under which the magnitude of variance difference and, therefore, the power to detect interaction is maximal. The value of the optimal effect depends on the interaction effect, variance of the factor and the variance of error term. As in our study variance of the factor and the variance of error term is fixed to one, the optimal effect of the factor depends on the interaction effect only. For simplicity, power was studied using the optimal main effect of the interacting factor. The range of the optimal effects of the factor used in this study can be found in the Additional file 4, Figure S1.

In both type I error and power estimation, the null hypothesis was rejected when threshold *p-*value ≤ 0.05 was reached.

### The VariABEL package

The VariABEL software implementing the SVLM is designed as an R package written in C++ and R languages. For regression analysis used by the SVLM method, the LAPACK functions "dgeqrf" and "ch2inv", which are part of the R distribution, were used. The package was compiled with gcc version 4.1.2 under Linux with version 2.6.18-274.7.1.el5 (Red Hat 4.1.2-51) and tested in R of version 2.13.1. The package is distributed under the GNU GPL license (v. 2.0 or later).

Stable version of the VariABEL package can be downloaded from the Comprehensive R Archive Network, CRAN [8] (http://www.r-project.org/). Installation is possible from R directly by running the command "install.packages("VariABEL")". Documentation is available as a part of the distribution and also on-line at the GenABEL project web-site (http://www.genabel.org). Developmental version of the package is available from the GenABEL project development pages (http://genabel.r-forge.r-project.org) located at R-forge [9].

The first stage of SVLM analysis consists of standard regression analysis which is used to access association between mean values of the trait and SNPs. VariABEL output contains results from both stages of analysis (modeling of means and variances). Thus, the VariABEL can be used for regular GWAS as well.

## Results and discussion

### Type I error and power

As it was mentioned above, we studied three different distributions of *ϵ*_*i*_. The SVLM test had acceptable type I error for all of them: *α*_normal _= 0.0471 ± 0.0015, $\alpha_{\chi_{df=5}^{2}}=0.0488\pm 0.00152$, and $\alpha_{\chi_{df=1}^{2}}=0.04955\pm 0.00153$ under fixed threshold *p *≤ 0.05. We did not see any significant deviation from nominal type I error rate of 5% for allele frequencies 5%, 40%, 60% and 95%. To understand the minimum sample size in a genotypic group under which the type I error of SVLM test still stays at a nominal level we measured type I error for allele frequencies 0.0005, 0.00075, 0.001, 0.002, 0.003, 0.004 and 0.005. These allele frequencies correspond to number of heterozygotes in a sample 10, 15, 20, 40, 60, 80 and 100. For those frequencies the type I errors (with its standard errors) were 0.028 ± 0.001, 0.032 ± 0.001, 0.037 ± 0.001, 0.042 ± 0.001, 0.044 ± 0.001, 0.049 ± 0.002 and 0.045 ± 0.002 correspondingly. This suggests that SVLM test has correct type I error rate when sample size in one of a genotypic group is not less then 80, while for smaller values the SVLM test starts being conservative. Levene's test did not show significant deviation from nominal value of 5% under these extremely low sample sizes.

**Figure 2:**
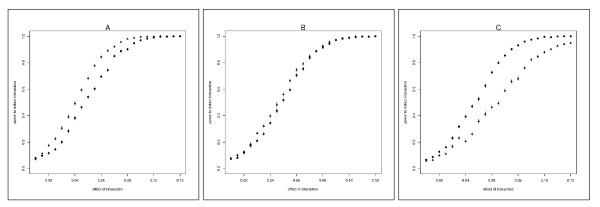
**Power to detect variance heterogeneity induced by interaction**. Power to detect variance heterogeneity at *p *≤ 0.05 using Levene's (circles) and SVLM (triangles) tests, as a function of the interaction effect, *β*_*gF*_. Interacting allele frequency is 0.4. (A) Error term *ϵ*_*i *_follows Normal distribution (B) Error term follows $\chi_{df=5}^{2}$ distribution (C) Error term follows $\chi_{df=1}^{2}$ distribution.

Figure 2 shows the dependence of the power of the SVLM (triangles) and the Levene's (circles) tests on the effect of interaction for differently distributed error term (*ϵ*_*i*_). Figure 2 shows that the power of the SVLM test depends on the skewness of the error term distribution stronger than the power of the Levene's test. When error term follows Normal distribution, the power of SVLM test is greater than the power of Levene's test (Figure 2, panel A). When the error term follows *χ*^*2 *^distribution with *df *= 5, the power of the SVLM test and Levene's test are similar (panel B). In case of higher skewness the SVLM test has lower power than the Levene's test (panel C). This can be explained by the fact that Levene's test is known to be robust to the deviations from normality, while the SVLM test is in fact a regression analysis for which the outcome is supposed to follow a normal distribution.

Additional file 1, Figure S1, Additional file 2, Figure S1 and Additional file 3, Figure S1 show the dependence of the power of the SVLM test and the Levene's test on the effect of interaction for different frequencies of the interacting allele.

As it is expected the power of both tests decreases when allele frequency decreases. The observation that SVLM's test power is affected by skewness more than the power of Levene's test stays true for all studied allele frequencies.

### Performance

The analysis by the SVLM test of 2543887 SNPs of 2715 subjects takes 46 minutes on one core of a Sun Fire X4540 Server with Quad-Core AMD Opteron Processor 2356.

## Discussion

Genome-wide association analysis is currently a primary tool for identification of loci associated with complex human traits. Testing for association under complex genetic models involving multiple interactions represents methodologically and computationally challenging task.

We and others have developed a method allowing testing of SNPs genome-widely for possible involvement into interaction [2,3] via testing of the heterogeneity of variance of the trait conditional on the genotype. Here we extend this method to imputed SNPs. The method we suggest, SVLM, is based on linear regression, and therefore results obtained in individual studies can be easily meta-analyzed using conventional methods and software tools.

Analysis of genotypic variances can be of interest to medical research. Assuming that there is a certain genotype associated with high variance of, for instance, blood pressure, the subjects having this genotype can be at risk of having extremely low or extremely high blood pressure.

In developing our method for analysis of variances using imputed data we have utilized the fact that the variance is, by definition, the expectation of squared values of the variable in case of zero mathematical expectation of this variable. This allowed us re-formulate the task of estimation and analysis of variances of the trait as a task of regression analysis of transformed trait. In this setting, methodological and computational tools developed for GWAS are applicable for the variance analysis.

The most important advantage of the proposed method is the possibility to detect SNPs belonging to a complex genetic network with many interacting factors that is impossible to study with standard tools. These SNPs will show variance heterogeneity and using our method these SNPs can be detected without knowing all the factors involved into this network. To find the factors, which interact with the identified SNP, a follow-up analysis can be applied where interaction between the SNPs found in variance analysis and all other measured SNPs or environmental factors are tested. In a case of interaction with an unknown factor, the SNPs showing significant variance differences still can be used to improve the variance explained as shown in the example below.

Consider a scenario in which SNPs, associated with a trait found in regular GWAS's, together explain a certain proportion of total trait's variance:

(4) $$R_{total}^{2}=\frac{\sigma_{GW\;AS}^{2}}{\sigma_{total}^{2}},$$

where $R_{total}^{2}$ is the proportion of total explained variance, $\sigma_{GW\;AS}^{2}$ is the variance explained by GWAS SNPs, and $\sigma_{total}^{2}$ is the trait's variance. In addition a SNP has been found by variance analysis, showing different genotypic variances in a way where presence of interacting allele *B *increases trait's variance: $\sigma_{AA}^{2}<\sigma_{AB}^{2}<\sigma_{BB}^{2}$, where $\sigma_{AA}^{2},\sigma_{AB}^{2}$, and $\sigma_{BB}^{2}$ are variances for the respective genotypes group *AA, AB*, and *BB*. Assume that allele frequencies and the effects of the SNPs found in GWAS and which contributed into $\sigma_{GW\;AS}^{2}$ are the same in each genotypic group *AA, AB*, and *BB *of the interacting SNP. Then the proportions of explained variance for different genotypic groups are:

$$\begin{array}{lll} R_{AA}^{2} & =\frac{\sigma_{GW\;AS}^{2}}{\sigma_{AA}^{2}}\; & \text{(1)}\\ R_{AB}^{2} & =\frac{\sigma_{GW\;AS}^{2}}{\sigma_{AB}^{2}}\; & \text{(2)}\\ R_{BB}^{2} & =\frac{\sigma_{GW\;AS}^{2}}{\sigma_{BB}^{2}}\; & \text{(3)}\\ & \; & \text{(4)} \end{array}$$

where $R_{AA}^{2},R_{AB}^{2},R_{BB}^{2}$ are proportions of variances explained by GWAS SNPs in individuals with genotypes *AA, AB*, and *BB*, respectively, at the SNP identified by the variance analysis. Taking into account that $\sigma_{AA}^{2}<\sigma_{AB}^{2}<\sigma_{BB}^{2}$ it follows that the proportions explained variance by the GWAS SNPs is higher in genotypic group *AA *compared to *AB *and *BB*, and higher in genotypic group *AB *compared to $BB:R_{AA}^{2}>R_{AB}^{2}>R_{BB}^{2}$. The value of the proportion of total explained variance $\left(R_{total}^{2}\right)$ is between $R_{AA}^{2}$ and $R_{BB}^{2}$ and this value depends on interacting allele frequency, effect of interaction, variance and effect of interacting factor. Thus, in such a scenario there is at least one genotypic group (AA) for which SNPs found in GWAS's explain more of the trait's variance $\sigma_{AA}^{2}$ compared to the total trait variance $\sigma_{total}^{2}$. To perform genotypic variance analysis for pedigree-based studies we propose to use GRAMMAR [10] implemented into GenABEL software [11]. In GRAMMAR the mixed model is applied where the trait is adjusted on random additive polygenic effect. Residuals from this model are free from polygenic familiar correlations and can be used for variance analysis.

To increase power of variance analysis by including the data from other studies the same approach as for regular GWAS can be used where the analysis is done for each cohort separately, followed by meta-analysis. The SVLM method can be used for discovering interacting SNPs following any of additive, dominant, recessive, over-dominant (where trait's variance among heterozygotes is increased), or genotypic models. In case of testing the additive variance model only, the SVLM test has maximal power in the case when the SNP follows true additive model and less power in case of dominant, recessive and over-dominant models. It is of interest to note that in case of over dominant model the power to detect interaction by the SVLM test is zero if the minor allele frequency (MAF) is 0.5 and increases with decreasing MAF. In a case when MAF is close to 0.5 Levene's test has higher performance.

## Conclusion

In this work we present further development of the method for detection of potentially interacting SNPs, extending it to the case of analysis of imputed SNPs. The method is based on testing of heterogeneity of trait's variance conditional on the genotype of locus being tested. We also present an R package, VariABEL, to facilitate for such analysis in genome-wide context. The package implements already existing variance heterogeneity tests, and the SVLM test developed in this work.

## Availability and requirements

**Project name**: VariABEL package

**Project home page**: http://www.genabel.org/packages/VariABEL

**Operating systems**: Linux, Mac OS X, Windows

**Programming language**: R, C++

**Other requirements**: R (≥ 2.13.0)

**License**: GNU GPL (≥ 2)

**Any restrictions to use by non-academics**: none except these posed by the license

## List of abbreviations

GWAS: Genome-Wide Association Study; MAF: Minor Allele Frequency; SNP: Single Nuclear Polymorphism, SVLM: Squared residual Value Linear Modeling.

## Competing interests

The authors declare that they have no competing interests.

## Authors' contributions

MS wrote the software, planned and carried out the simulation study and wrote the manuscript. NA, PE, CvD and YA planned the simulation study and wrote the manuscript. All authors read and approved the final manuscript.

## Supplementary Material

Additional file 1 **Power to detect variance heterogeneity induced by interaction, assuming Normal distribution of residual error**. The file contains the figure describing dependency of power to detect variance heterogeneity induced by interaction and the effect of interaction, *β*_*gF*_, using Levene's (circles) and SVLM (triangles) tests. The residual error follows Normal distribution. Scenarios with different frequencies of interacting allele are given in Panel A - 5%, Panel B - 40%, Panel C - 60%, and Panel D - 95%. Click here for file

Additional file 2 **Power to detect variance heterogeneity induced by interaction, assuming **$\chi_{df=5}^{2}$**distribution of residual error**. The file contains the figure describing dependency of power to detect variance heterogeneity induced by interaction and the effect of interaction, *β*_*gF*_, using Levene's (circles) and SVLM (triangles) tests. The residual error follows $\chi_{df=5}^{2}$ distribution. Scenarios with different frequencies of interacting allele are given in Panel A - 5%, Panel B - 40%, Panel C - 60%, and Panel D - 95%. Click here for file

Additional file 3 **Power to detect variance heterogeneity induced by interaction, assuming $\chi_{df=1}^{2}$ distribution of residual error**. The file contains the figure describing dependency of power to detect variance heterogeneity induced by interaction and the effect of interaction, *β*_*gF*_, using Levene's (circles) and SVLM (triangles) tests. The residual error follows $\chi_{df=1}^{2}$ distribution. Scenarios with different frequencies of interacting allele are given in Panel A - 5%, Panel B - 40%, Panel C - 60%, and Panel D - 95%. Click here for file

Additional file 4 **Optimal effect of the factor *F *(*β***_***F***_**) as a function of the interaction effect (***β*_***gF***_**)**. The file contains the figure showing the value of optimal effect of interacting factor *F, β*_*F*_, as a function of the effect of interaction, *β*_*gF *_for allele frequencies 5% (black), 40% (red), 60% (green) and 95% (yellow). Click here for file
