# Supplementary material for: An R package "VariABEL" for genome-wide searching of potentially interacting loci by testing genotypic variance heterogeneity
Source: BMC Genet. 2012 Jan 24;13:4. doi: 10.1186/1471-2156-13-4 (PMC3398297; doi:10.1186/1471-2156-13-4)
Supplement: Additional file 1 — Power to detect variance heterogeneity induced by interaction, assuming Normal distribution of residual error. The file contains the figure describing dependency of power to detect variance heterogeneity induced by interaction and the effect of interaction, βgF, using Levene's (circles) and SVLM (triangles) tests. The residual error follows Normal distribution. Scenarios with different frequencies of interacting allele are given in Panel A - 5%, Panel B - 40%, Panel C - 60%, and Panel D - 95%. [file 1471-2156-13-4-S1.PDF]

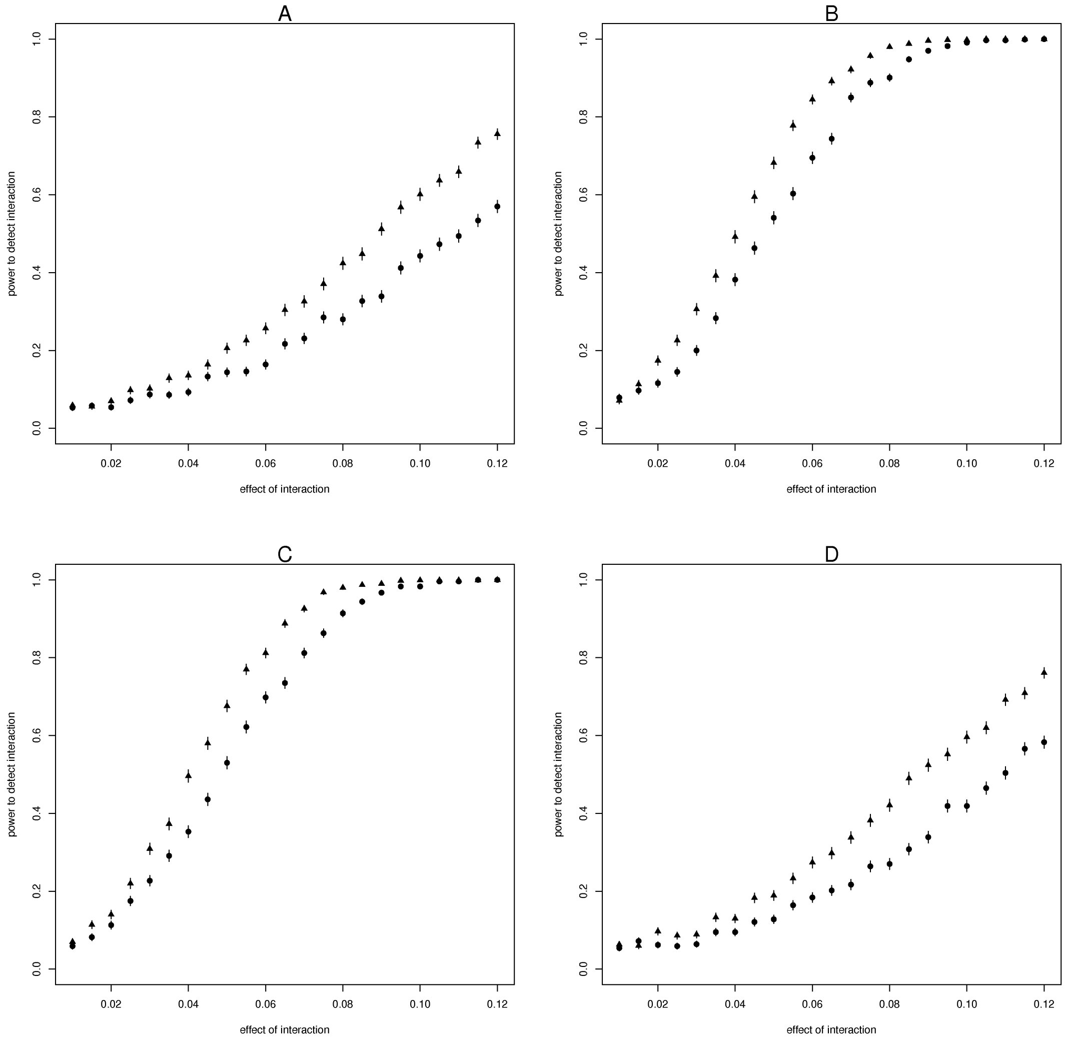

Figure S1: **Power to detect variance heterogeneity induced by interaction, assuming Normal distribution of residual error.** Dependency of power to detect variance heterogeneity induced by interaction and the effect of interaction, β_{gF}, using Levene's (circles) and SVLM (triangles) tests. The residual error follows Normal distribution. Scenarios with different frequencies of interacting allele are given in Panel A – 5%, Panel B – 40%, Panel C – 60%, and Panel D – 95%.
