# Supplementary material for: An R package "VariABEL" for genome-wide searching of potentially interacting loci by testing genotypic variance heterogeneity
Source: BMC Genet. 2012 Jan 24;13:4. doi: 10.1186/1471-2156-13-4 (PMC3398297; doi:10.1186/1471-2156-13-4)
Supplement: Additional file 4 — Optimal effect of the factor F (βF) as a function of the interaction effect (βgF). The file contains the figure showing the value of optimal effect of interacting factor F, βF, as a function of the effect of interaction, βgF for allele frequencies 5% (black), 40% (red), 60% (green) and 95% (yellow). [file 1471-2156-13-4-S4.PDF]

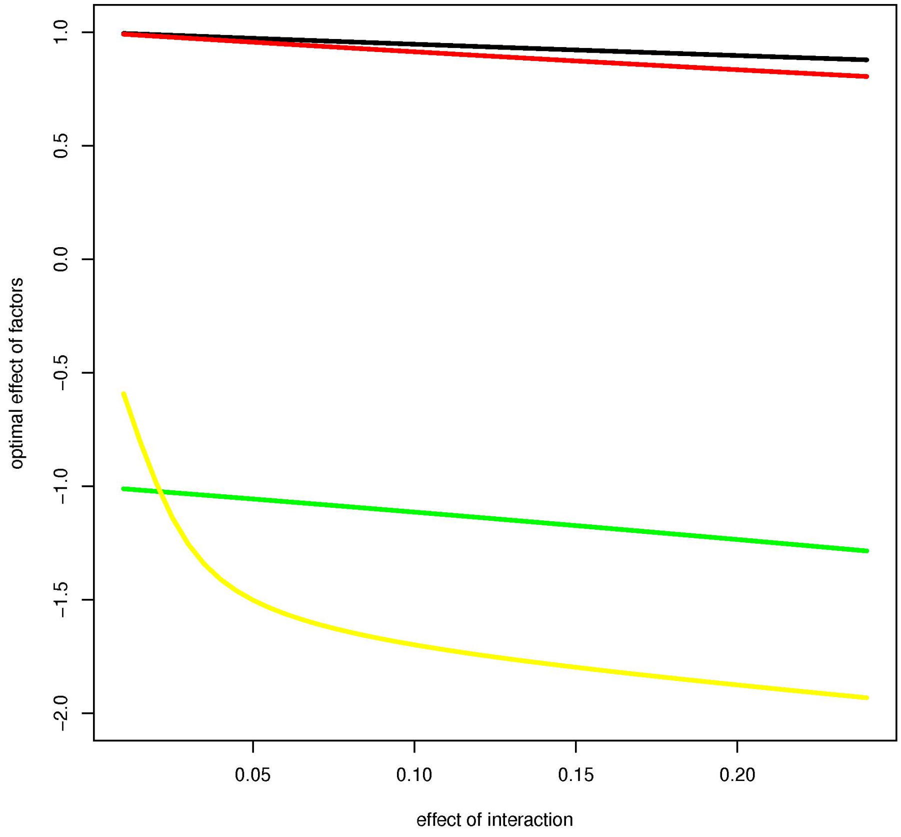

Figure S1: **Optimal effect of the factor F (β_F) as a function of the interaction effect (β_{gF}).** The value of optimal effect of interacting factor F, β_F, as a function of the effect of interaction, β_{gF} for allele frequencies 5% (black), 40% (red), 60% (green) and 95% (yellow).
